# The implications of the diving response in altering carbon dioxide sensitivity as measured by changes in heart rate, respiration rate and psychological measures in panic disorder patients

**DOI:** 10.3389/fpsyt.2025.1533019

**Published:** 2025-06-02

**Authors:** Peter Kyriakoulis, Catherine Lissette Caballero

**Affiliations:** ^1^ School of Health Sciences, Faculty of Health, Arts and Design, Swinburne University of Technology, Melbourne, VIC, Australia; ^2^ School of Psychology, Faculty of Health, Deakin University, Melbourne, VIC, Australia

**Keywords:** panic disorder, anxiety, diving response, cold facial immersion, carbon dioxide sensitivity

## Abstract

**Introduction:**

Breath-hold divers are known for their exceptional breathing control and reduced carbon dioxide (CO_2_) sensitivity due to training adaptations. In contrast, individuals with panic disorder (PD) often exhibit heightened CO_2_ sensitivity. This study aimed to explore the potential clinical applications of the diving response (DR), particularly cold facial immersion (CFI), in mitigating panic-related symptoms and cognitions by modulating CO_2_ sensitivity.

**Methods:**

This study investigated the effects of the CFI task on individuals with PD and a comparison group. Changes in heart rate, respiration rate, and psychological measures were assessed before and after a CO_2_ challenge to determine whether the CFI task could reduce CO_2_ sensitivity and panic-related symptoms.

**Results:**

The results did not support the efficacy of the CFI task in reducing physiological markers of CO_2_ sensitivity—specifically, heart rate and respiration rate—following the CO_2_ challenge in either the clinical or comparison group, potentially due to the small sample size. However, significant reductions in both physiological and cognitive symptoms of panic were observed in the clinical group following the CFI task.

**Discussion:**

As hypothesized, the CFI task demonstrated anxiolytic effects in individuals with PD by reducing self-reported anxiety and panic symptoms. These findings highlight the potential of the CFI task for clinical application in the treatment of panic disorder, warranting further research with larger samples.

## Introduction

Panic disorder (PD) is a debilitating psychiatric condition marked by spontaneous, recurrent panic attacks, affecting approximately 4% of the general population and leading to various personal and socioeconomic challenges (1). Individuals with PD display a thoracic breathing pattern characterized by abnormal variability and irregularity (2). They often experience cardiorespiratory symptoms such as air hunger, dyspnea, rapid breathing, and increased heart rate (3).

Increased carbon dioxide (CO_2_) sensitivity is a common feature among individuals with PD, and is frequently observed in the respiratory subtype of PD (4). The CO_2_ hypersensitivity theory suggests that individuals with PD have a lower threshold for detecting CO_2_ levels (5). This supports the presence of an evolved suffocation alarm system that helps the brain monitor available oxygen (6). For individuals with PD, the 35% CO_2_ challenge (CO_2_ challenge), a single-breath inhalation of a gas mixture containing 35% CO_2_ and oxygen (O_2_), is commonly used to induce an exaggerated physiological and psychological response that mimics a panic attack. This method helps assess CO_2_ sensitivity and autonomic reactivity in PD research (6–11). The central and peripheral chemoreflexes are activated during the CO_2_ challenge, leading to increased ventilation in an effort to remove excess CO_2_ and stabilize blood pH. This response and the anxiogenic effects of the CO_2_ challenge may be more sensitive in individuals with PD (12).

Freedivers who practice breath-hold diving, relying on a single deep breath without an external air supply, have been practicing this ancestral form of diving since ancient times (13). Freedivers are a unique group of individuals that have been known for their exceptional breathing control, lower CO_2_ sensitivity, and pronounced diving response (DR) due to trained effects (14–16). The DR is a physiological reflex that optimizes respiration, allowing humans to endure a lack of oxygen underwater (14). The DR triggers the peripheral chemoreflex which is activated by cold water on the face, leading to vasoconstriction and bradycardia to conserve oxygen. While the DR initially focuses on oxygen conservation, the central chemoreflex is also stimulated and becomes more prominent as CO_2_ builds up (17). Daily breath-hold training for as little as two weeks has been demonstrated to enhance breath-hold duration and trigger the diving DR more quickly (15). After breath-hold training, apneas of the same length led to less arterial oxygen desaturation, suggesting improved oxygen conservation due to the pronounced effect of the DR, an innate adaptation that can be trained (18). Reduced chemosensitivity to hypercapnia has been observed in groups of freedivers, including synchronized swimmers, underwater hockey players, and trained young competitive swimmers. This suggests that sub-aquatic training involving repetitive breath-holding may lead to desensitization of peripheral chemoreflexes (13).

While both freedivers and individuals with PD exhibit altered CO_2_ sensitivity, the patterns are opposite. Freedivers have reduced central chemosensitivity and normal peripheral chemosensitivity, enabling prolonged breath-holding. In contrast, those with PD tend to have increased central chemosensitivity, leading to an exaggerated response to CO_2_ and potentially contributing to panic symptoms (12). In their study, Kyriakoulis et al. (12) discovered that the CO_2_ challenge triggered anxiety and panic symptoms in clinical participants. In contrast, the cold facial immersion (CFI) task exhibited anxiolytic effects, as evidenced by a reduction in heart rate and a decrease in self-reported symptoms of anxiety and panic in both clinical and comparison groups. The findings of the current study support this hypothesis, revealing that both Clinical and Comparison participants experienced a significant bradycardic effect, with a decrease of approximately 30–35 beats per minute, following the CFI task (12).

This study aimed to investigate whether the cold facial immersion task alters one’s physiological and psychological response to the CO_2_ challenge. Additionally, the study aims to explore whether the CFI task can serve as an intervention to alleviate and prevent panic symptoms and panic attacks. It is noteworthy to recognize that by activating the DR and lowering heart rate, individuals may experience a reduction in both physiological and cognitive symptoms of panic, as well as a possible decrease in sensitivity to CO_2_ (12). Furthermore, the rationale for this study appears to follow a coherent progression, as existing literature suggests that individuals with PD exhibit heightened sensitivity to CO_2_ (19, 20). Additionally, the findings from a previous study by Kyriakoulis et al. (12) demonstrated that the CFI task reduced both physiological and cognitive symptoms of anxiety, hence it was conceptualized that the activation of the DR may be responsible for altering CO_2_ sensitivity in individuals with PD, a topic that warrants further investigation. In designing this study, the researchers wanted to establish whether trained effects of the CFI task could change individuals’ response to the CO_2_ challenge.

Consequently, an experimental protocol was developed to explore this further. It was hypothesized that CFI would reduce Clinical and Comparison participants’ sensitivity to CO_2_ as evidenced by heart rate and respiration rate reductions following the subsequent CO_2._ It was further hypothesized that participants in both groups would report a reduction in anxiety symptoms following CFI and subsequent CO_2_ when compared to pre-measures and the CO_2_ administration without CFI.

This study is the first attempt to examine whether the activation of the DR via CFI may alter CO_2_ sensitivity in PD individuals. This study aims to further investigate whether CFI has a preventative effect on panic-related symptoms and cognitions. This study examines the effect of CFI in altering CO_2_ sensitivity in participants with PD.

## Method

### Participants and sampling

Investigations were carried out with 30 participants: 15 patients with a primary diagnosis of PD with or without agoraphobia (DSM-5, in the Clinical Group, and 15 healthy comparisons in the Comparison Group, who did not meet the criteria for PD or mental illness. Of the 30 participants, 15 were male, and 15 were females. The participants in the Clinical Group had an average age of 36.3 years (SD = 13.8), whilst the participants in the Comparison group had an average age of 33.1 years (SD = 7.7). Both groups comprised 6 males and 9 females. Difficulties with recruitment resulted in the participants not being able to be matched for age and gender and five clinical participants from the preliminary study were invited to join the Clinical Group for this study. Although attempts were made in Study 2 to match participants and to recruit non-university students as comparisons, this was difficult to accomplish. Sample size calculations were not possible as the response to CO_2_ and CFI has not previously been investigated among persons with PD. Therefore, we recruited as many participants as possible within the time and budget constraints of the research study. The cohort differences are reported in the results section.

Health screening assessments were carried out by a medical doctor (at Swinburne University) to establish medical eligibility to undergo the CO_2_ challenge. As part of the health screening, all participants completed a demographic and health information form. Demographical data such as age, gender, level of education completed, employment status were included in this form. In addition, the demographical questionnaire included a health screening, height and weight information, as well as fitness and exercise assessment which included a physical activity index. Participants were also asked about their perceived level of comfort with water.

The Mini-International Neuropsychiatric Interview (MINI) is a short structured diagnostic interview used to make diagnoses of Axis I disorders (DSM-IV) and has demonstrated high reliability and validity (21). It was used to screen psychological disorders within the exclusion criteria and identify individuals with PD (DSM-IV). Exclusion criteria for both groups included psychotic disorders, substance abuse, prescription medication, habitual use of benzodiazepines, known allergies to latex, asthma or respiratory problems, cardiovascular problems, hypertension, hypotension, pregnancy, and cerebrovascular problems including epilepsy and organic brain disorder. Finally, other comorbidities to Axis I mental health disorders (DSM-IV) were excluded with the exception of Depression, Generalized Anxiety Disorder and Social Anxiety Disorder, if secondary to PD, along with those with biological relatives with PD.

The Clinical Group was also assessed with the structured clinical interview (SCID-I) for the DSM-IV Axis I Disorders module for PD and Agoraphobia (SCID-I) (22). The SCID-I is a comprehensive structured interview for the diagnosis of psychiatric disorders according to DSM-IV criteria (23). To encourage higher participation rates among PD participants, two movie tickets were offered to each applicant who completed the study.

### Physiological measures

A compact physiological monitoring system (Zephyr Bioharness) featuring a chest strap and external multi-recording and monitoring device was used to measure heart rate, posture and respiration rate. Participants were connected to a Zephyr Bioharness and measured throughout the experimental study. Participants’ breath-hold ability was also measured during the experimental phase and included breath-hold ability after maximum exhalation and breath-hold ability following maximum inhalation. All participants undertook the conditions of the experimental study while wearing the chest strap connected to the Zephyr Bioharness, which was connected via Bluetooth to a multirecording and monitoring device (PowerLab). PowerLab version 7.0 data acquisition and analysis software were used as a multirecording device, and recorded data were sampled at a frequency of 60 Hz (60 samples per second). Physiological measures, including heart rate and respiration rate, were recorded at Time 2 and Time 3 of the study.

### Measures

All participants were required to complete a battery of psychological assessments at three time points throughout the study. **Table 1** provides a list of the battery of psychological measures and the time points at which the measures were administered throughout the study.

**Table 1:** Battery of psychological assessments administered before and during the study.

Cognitive Measures	Administrations
1. Anxiety Sensitivity Index (ASI)	Time 1 (Baseline), Time 2, Time 3
2. Beck Anxiety Inventory (BAI)	Time 1 (Baseline), Time 2, Time 3
3. Discomfort Intolerance Scale (DIS)	Time 1 (Baseline)
4. State/Trait Anxiety Inventory (STAI) (A-Trait)	Time 1 (Baseline)
5. State/Trait Anxiety Inventory (STAI) (A-State)	Time 1 (Baseline), Time 2, Time 3
6. Panic Attack Cognitions Questionnaire (PACQ)	Time 1 (Baseline), Time 2, Time 3
7. Acute Panic Inventory (API)	Time 1 (Baseline), Time 2, Time 3
8. Visual Analog Scale for Anxiety (VAS) (Participant & Researcher)	Time 2, Time 3 (x 2)
9. Center for Epidemiological Studies Depression Scale (CES-D)	Time 1 (Baseline)

### Research design

**Figure 1:**
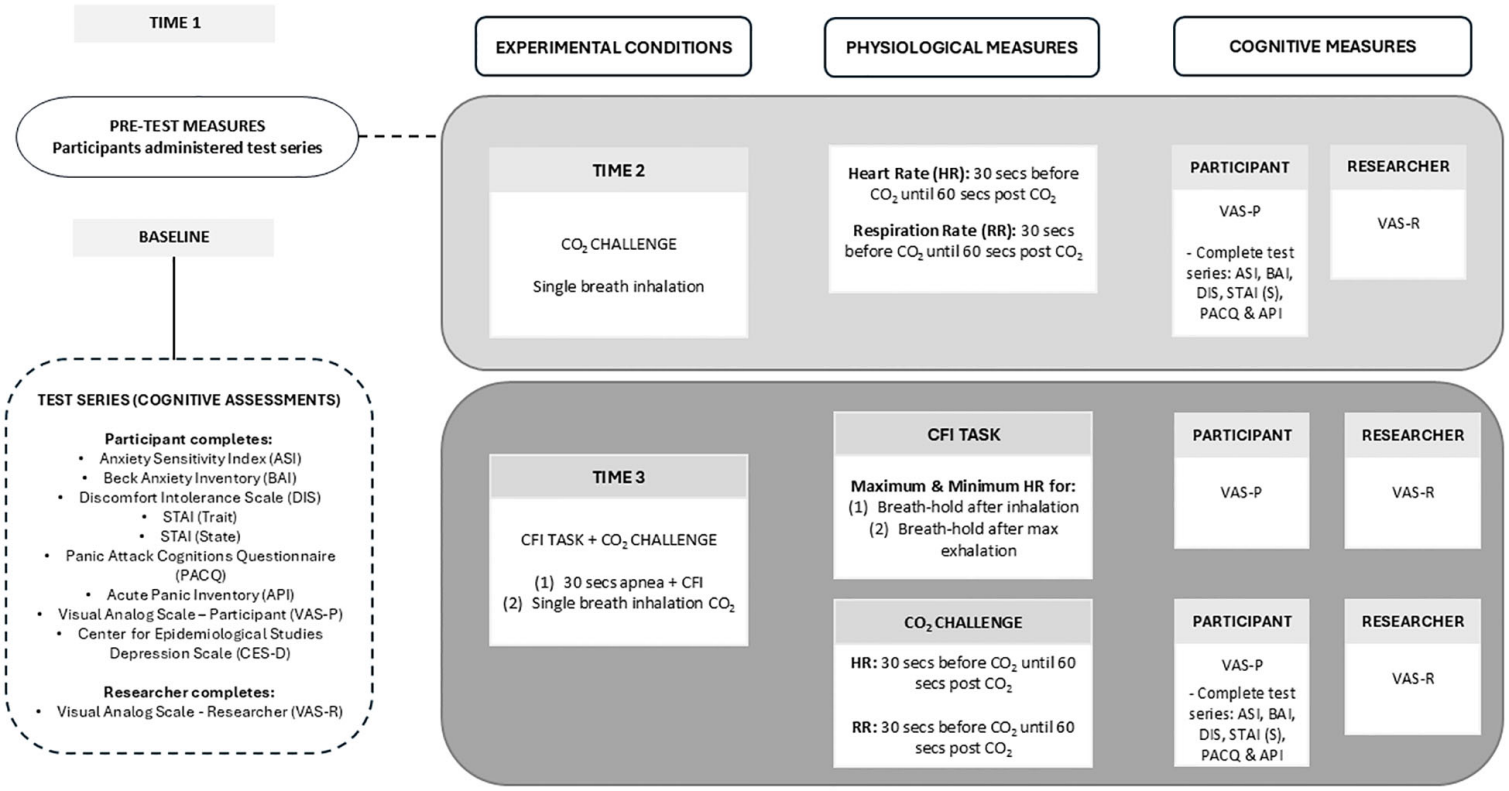
Experimental procedure for data collection in the clinical and comparison groups.

Participants took part in two experimental challenges at two different time intervals, including the (1) CO_2_ Challenge, and (2) CFI Task followed by CO_2_ Challenge. **Figure 1** displays the experimental procedure indicating when physiological and cognitive measures were collected as part of this study.

In the CO_2_ Challenge, participants were instructed to take one maximum inhalation of a 35% CO_2_ and 65% O_2_ mixture, hold their breath for 4 seconds, and then exhale. In the CFI Task, participants were instructed to take a deep breath and immerse their face in a tub filled with cold water regulated between 7 ˚C - 12˚C, whilst maintaining the room temperature at a constant 22˚C.

### Procedure

The experimental study was held at a clinical office at Swinburne University, which was specifically set up for the research. Experimental conditions were not randomly assigned to the Clinical and Comparison participants, who were required to undertake Time 1 and Time 2 procedures on the same day, either in the morning or afternoon. Participants were scheduled to complete Time 3 tasks on a different day, as ethics approval only allowed for participants to complete one CO_2_ challenge per day. Participants were asked to refrain from caffeine-containing beverages, including cola and smoking, as well as physical exercise for at least two hours prior to the CO_2_ challenge. They were also asked to refrain from alcohol from the day prior. Testing was conducted in the morning or the afternoon.

### Data cleaning

Prior to statistical analyses, all variables were assessed for the presence of missing data and outliers. The data revealed missing values and hence Little’s Missing Completely at Random (MCAR) test was used to assess whether data were missing at random. Assumptions for the MCAR test were assessed and fulfilled, χ^2^ (39) = 46.165, DF = 39, p >.005. Missing values were replaced using the expectation-maximization (EM) algorithm for imputation. Prior to conducting statistical analyses, the distributions of all variables were visually inspected to determine if they met the assumption of normality of distribution, a requirement of parametric statistical analyses. In cases where data did not adequately meet the assumptions of normality and could not be transformed to normalize their distributions according to recommended procedures (24), non-parametric analyses were conducted.

### Statistical analyses

Prior to conducting parametric analyses, data were inspected to ensure they met the assumptions for such analyses. Physiological data, including respiration rate and heart rate data satisfied the assumptions for parametric analysis. Hence t-tests and ANOVA analyses were employed to investigate the differences between the Clinical and Comparison Groups on the experimental conditions. Examination of normal distribution revealed that scores across all self-report cognitive measures taken at Time 1(Pre-test), at Time 2 (CO_2_) and Time 3 (CFI followed by CO_2_), including API, ASI, BAI, PACQ, STAI, VAS-R, and VAS-P did not meet the assumptions of normal distribution for parametric analyses. Hence non-parametric tests were utilized. Spearman rank-order correlation coefficient was used to investigate the relationships between the psychological measures. Friedman’s test was used to examine differences in the cognitive measures collected across the experimental conditions. Demographic information was compared between the Clinical Group and the Comparison Group using chi-squared tests of association and Fishers. The Statistical Package for Social Sciences version 22.0 was used for all analyses.

## Results

### Overview of analysis

The analysis of this study is presented in three sections. The first section presents the comparison of demographic details between groups and the correlations of the Time 1 (Pre-test) measures. The second section reports an examination of the physiological differences between the Clinical and Comparison Groups at Time 1 and 2. This section also reports the results of mixed ANOVAs, examining the effects of Time 1 and Time 2 tasks on participant’s physiological responses (heart rate and respiration rate). The third section examines the effects of the CO_2_ challenge and ultimately the effect of CFI on CO_2_ sensitivity across the anxiety measures. When outcomes were not normally distributed, Friedman tests were used instead of ANOVA tests and Wilcoxon signed–rank tests were used instead of paired t-tests.

### Correlations between premeasures

**Tables 2** and **3** provide a comparison for the Clinical and Comparison Groups. No significant differences were found. In examining the demographic information in Study 2, the main differences included fewer participants in the Clinical Group who reported drinking alcohol. The mean average age for the Clinical Group (M = 36.33, SD = 13.77), and the Comparison Group (M = 33.13, SD = 7.71) respectively.

**Table 2:** Demographic information categorical data.

Variable	Category	Clinical Group		Comparison Group	
		N	%	N	%
Gender	Fisher’s exact test, *p* = 1.000				
	Male	6	40.00	6	40.00
	Female	9	60.00	9	60.00
Age	Fisher’s exact test, *p* = .168				
	20 – 29	7	46.67	6	40.00
	30 – 39	1	6.67	5	33.33
	40 – 60	7	46.67	4	26.67
Education Level	Fisher’s exact test, *p* = .005				
	No University Degree	9	60.00	1	6.67
	University Degree	6	40.00	14	93.33
Employment Status I	Fisher’s exact test, *p* = 1.000				
	Employed	8	53.33	7	46.77
	Unemployed/Student	7	46.77	8	53.33
Smoking	Fisher’s exact test, *p* = .651				
	Yes	4	26.67	2	13.33
	No	11	73.33	13	86.67
Drinking	Fisher’s exact test, *p* = .050				
	Yes	7	46.77	13	86.67
	No	8	53.33	2	13.33
Physical Fitness	Fisher’s exact test, *p* = .1000				
	Poor/Fair	5	25.00	6	40.00
	Good/Very Good	10	75.00	9	60.00
Physical Activity at Work	Fisher’s exact test, *p* = .715				
	Sedentary	9	60.00	7	46.77
	Non-sedentary	6	40.00	8	53.33
Employment status II	Fisher’s exact test, *p* = 1.000				
	Unemployed	5	25.00	1	6.67
	Employed/Student	10	75.00	14	93.33
Weekly Physical Exercise	Fisher’s exact test, *p* = .206				
	< 3 hrs	10	75.00	11	73.33
	>3 hrs	5	25.00	4	26.67
Weekly Cycling	Fisher’s exact test, *p* = 1.000				
	None	15	100.00	14	93.33
	Some	0	0.00	1	6.67
Weekly Walking	Fisher’s exact test, *p* = 1.000				
	< 3 hrs	7	46.77	8	53.33
	>3 hrs	8	53.33	7	46.77
Weekly home duties	Fisher’s exact test, *p* = 1.000				
	< 1 hr	9	60.00	11	50.0
	>3 hrs	6	40.00	4	50.0
Weekly gardening	Fisher’s exact test, *p* = .035				
	None	8	53.33	14	93.33
	Some	7	46.77	1	6.77
Walking Pace	Fisher’s exact test, *p* = 1.000				
	Slow/Steady, average	6	40.00	5	25.00
	Brisk pace/Fast > 6km	9	60.00	10	75.00

N = 30 (Clinical Group: *n* =15, Comparison Group: *n* = 15). p-values from Fisher’s exact test (2-sided).

**Table 3:** Demographic information continuous data.

Variable	Clinical Group		Comparison Group	
	M	SD	M	SD
Water Comfort (0 = Not at all comfortable - 10 = Very comfortable)	7.70	1.87	7.40	2.82
Average No. of Glasses per week (Alcohol)	2.23	3.42	2.73	2.93
No. Push Ups	9.93	6.64	15.73	14.42
Height	168.07	7.56	169.40	11.21
Weight	71.40	11.78	71.33	22.96

P-values for the Mann-Whitney U test (two-sided) are as follows: Water Comfort = .933, Average No. of Glasses per Week = .270, No. Push Ups = .443, Height = .406, Weight = .604.

The Spearman rank-order correlation coefficient was used as a non-parametric measure to determine the strength and direction of association that exists between the premeasure assessments. Correlation coefficients between premeasure assessments are presented in **Table 4**.

**Table 4:** Correlations between time 1 (premeasure) assessments.

Premeasures	PACQ	BAI	CESD	STAI-T	ASI	STAI_S	API	DIS
PACQ	1.00	.865**	.760**	.847	.792**	.734**	.656	.405
BAI		1.00	.719**	.744**	.813**	.602	.663	.422
CESD			1.00	.847**	.798**	.734**	.486	.219
STAI-T				1.00	.745**	.756**	.678	.164
ASI					1.00	.734	.559	.331
STAI-S						1.00	.726**	.313
API							1.00	.403
DIS								1.00

*N* = 30. **p <.001.

A series of t-tests for independent groups was employed to determine if significant differences existed between the clinical and the comparison group on the scores of all psychological measures at the Time 1 (Pre-test) stage. The results of the analysis indicated that there were significant differences (p <.05) across all psychological measures at Time 1 (Pre-test) except for the DIS which was not significant at (p >.05).

### Physiological differences at Time 2 (CO_2_ Challenge) and Time 3 (CFI + CO_2_ Challenge)

A mixed, ANOVA was conducted to compare the Clinical and Comparison Groups in terms of the effect of the CO_2_ challenge task and group on participants’ respiration rate. **Table 5** shows respiration rate means and standard deviations for all participants before and after the CO_2_ Challenge (Time 2) and before and after the CFI + CO_2_ Task (Time 3). In the tables and figures below “b” refers to “before” and “p” for “post”.

**Table 5:** Descriptive statistics for respiration rate (RR) before and after time 2 (CO_2_) and before and after time 3 (CFI + CO_2_).

RR Intervals	Group			
	Clinical (*n* = 15)		Comparison (*n* = 15)	
	*M*	*SD*	*M*	*SD*
RR Time 2 (b. CO_2)_	14.60	3.08	15.90	3.59
RR Time 2 (p. CO_2)_	15.30	3.03	16.95	3.59
RR Time 3 (b. CFI + CO_2_)	14.40	2.64	15.52	4.30
RR Time 3 (p. CFI + CO_2_)	14.34	2.41	16.32	4.21

There was no significant interaction between the effects of group and CO_2_ challenge task on participant’s respiration rate, (*F* = (1, 28) = .706, *p* = 0.38, η2 =.005). Simple main effects analysis showed that between 30 seconds prior to the CO_2_ challenge (Time 2) and 60 seconds following the CO_2_ challenge (Time 3), participants experienced no significant change in respiration rate, (*F* = (1, 28) = 3.549, *p* =.070, η2 =.112). There were no significant differences in respiration rates observed between the Clinical and Comparison Groups, (*F*(1, 30) = 1.704, *p* = .20, η2 =.057).

A mixed, ANOVA was also conducted to compare the Clinical and Comparison Groups in terms of the effect of the CO_2_ challenge task and group on participants’ HR. **Table 6** shows respiration rate means and standard deviations for all participants before and after the CO_2_ Challenge (Time 2) and before and after the CFI + CO_2_ Task (Time 3).

**Table 6:** Descriptive statistics for heart rate (HR) before and after time 2 (CO_2_) and after time 3 (CFI + CO_2_).

HR Intervals	Group			
	Clinical (*n* = 15)		Comparison (*n* = 15)	
	*M*	*SD*	*M*	*SD*
HR Time 2 (b. CO_2)_	95.37	16.02	92.86	22.58
HR Time 2 (p. CO_2_)	97.96	20.79	89.96	26.80
HR Time 3 (b. CFI + CO_2_)	100.31	19.08	87.84	19.59
HR Time 3 (p. CFI + CO_2_)	92.92	10.97	90.29	27.33

Furthermore, no significant interaction was found between the effects of group and CO_2_ on participant’s HR, (*F* = (1, 28) =1.028, *p* =.319, η2=.035). Simple main effects analysis showed that between 30 seconds prior to the CO_2_ challenge and 60 seconds following the CO_2_ challenge at Time 2, participants experienced no significant change in heart rate, (*F* = (1, 28) = .003, *p* = .955, η2=.000). There were also no significant differences between heart rates observed between the Clinical and Comparison Groups, (*F*(1, 28) = .489, *p* = .490, η2=.017).

**Figure 2:**
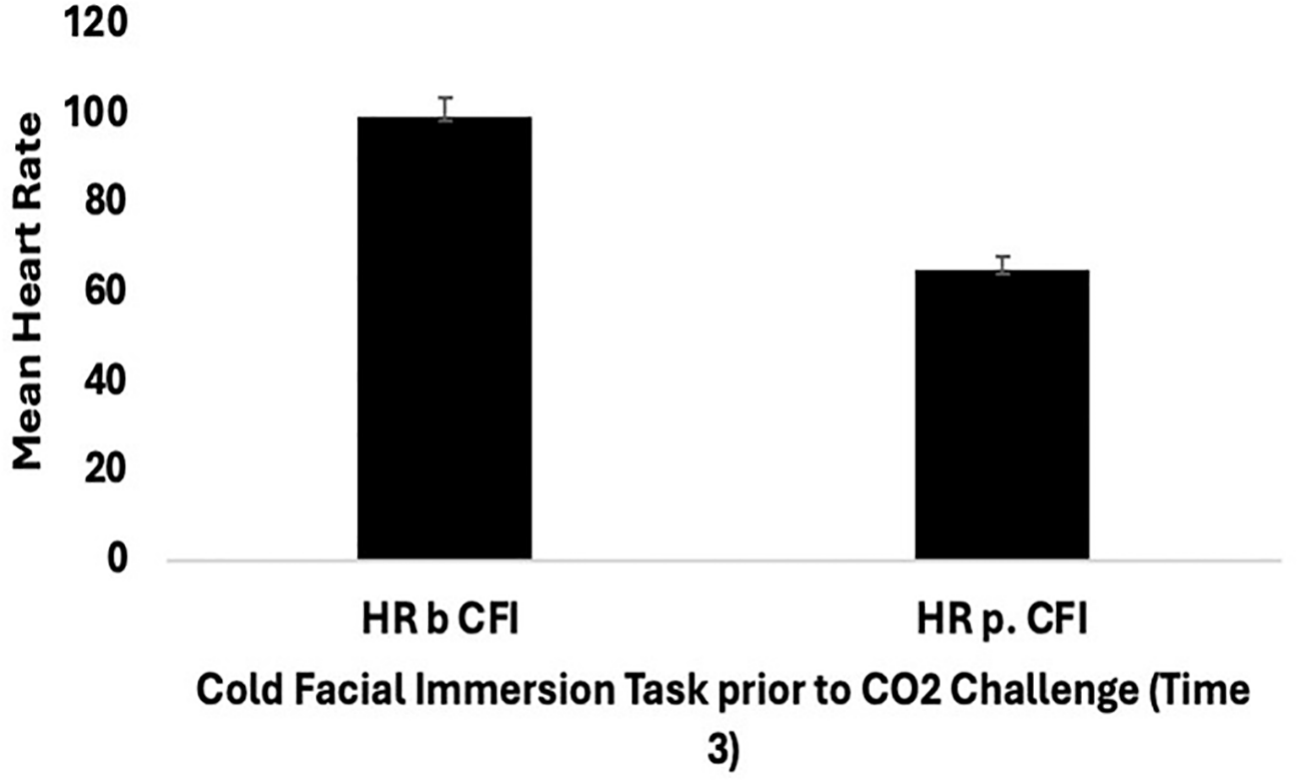
Mean heart rate (SE) for all participants (clinical group n = 15, and comparison group n = 15) pre and post cold facial immersion (CFI) prior to CO_2_ at Time 3.

Further mixed ANOVA analyses were conducted to compare the groups in terms of the effects of CFI, specifically at Time 2 (CO_2_ challenge) and Time 3 (CFI followed by the CO_2_ challenge), on participants’ heart rate (HR). A mixed ANOVA was conducted with the Clinical and Comparison Groups to examine the effect of CFI on participants’ heart rate and examine whether differences between groups were observed. There was no significant interaction between the effects of group and CFI on participant’s HR, (*F*(1, 28) = .074, *p* = .787, η2=.003). Simple main effects analysis showed that, at Time 3- both at the start and end of the CFI task (prior to the CO_2_ Challenge) - participants experienced a significant reduction in heart rate, (*F*(1, 28) = 121.492, *p* < 0.01, η^2^ = .813) (see **Figure 2**). However, no significant differences were observed between the Clinical and Comparison Groups, (*F*(1, 28) = 2.902, *p* = .100, η2 =.094).

### Cognitive measures and effect of CFI on CO_2_ sensitivity

**Table 7** provides means for all self-report cognitive measures taken at Time 1 (Pre-measures), Time 2 (CO_2_), and Time 3 (CFI followed by CO_2_).

**Table 7:** Medians, minimum, maximum and interquartile ranges for cognitive measures at Time 1, Time 2 and Time 3.

Cognitive Measures	Mdn	Min	Max	Interquartile Range
Acute Panic Inventory				
Time 1 (Pre-test) Time 2 (p. CO_2_) Time 3 (p.CFI + CO_2_)	1 7.5 2.5	0 0 0	31 42 23	2.25 13.25 5.25
Anxiety Sensitivity Index				
Time 1 (Pre-test) Time 2 (p. CO_2_) Time 3 (p. CFI + CO_2_)	33.5 34 27	17 16 17	73 80 63	28 27.5 24
Beck Anxiety Inventory				
Time 1 (Pre-test) Time 2 (p. CO_2_) Time 3 (p. CFI + CO_2_)	11.5 19.5 6	0 0 0	61 62 34	23.75 21.5 11.25
Panic Cognitions Questionnaire				
Time 1 (Pre-test) Time 2 (p. CO_2_) Time 3 (p. CFI + CO_2_)	10.5 4 2	0 0 0	68 75 27	31 18 12.25
State Anxiety Inventory				
Time 1 (Pre-test) Time 2 (p. CO_2_) Time 3 (CFI + CO_2_)	28.5 39.5 30	20 20 20	61 74 68	21.25 28.25 20.5
Visual Analog Scale – Participant (VAS-P)				
Time 1 (p. CO_2_) Time 2 (p. CO_2_ b. CFI) Time 3 (p. CFI + CO_2_)	5 0.5 3	0 0 0	10 5 8	6 2.13 4.13
Visual Analog Scale – Researcher (VAS-R)				
Time 1 (p. CO_2_) Time 2 (p. CO_2_ b. CFI) Time 3 (p. CFI + CO_2_)	5 0 2.5	0 0 0	10 4 8	5.5 2 3

N = 30 (Clinical Group: *n* =15, Comparison Group: *n* = 15).

**Figure 3:**
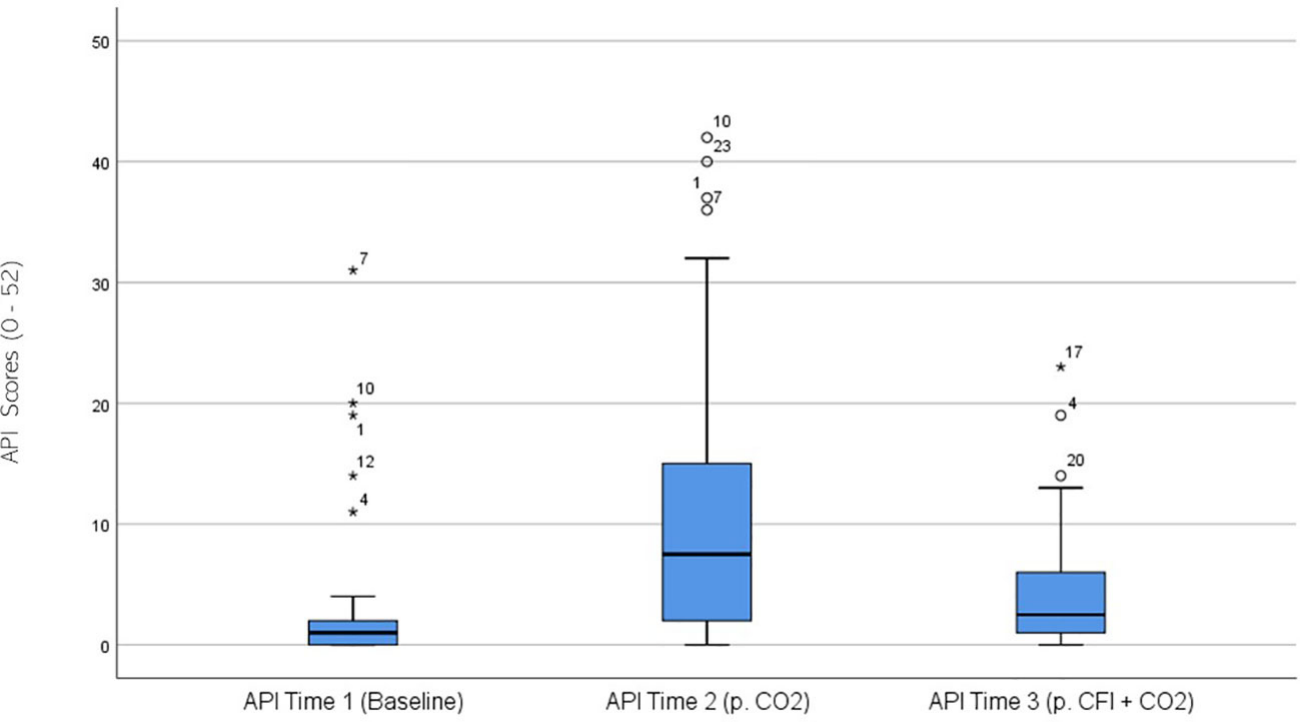
Box plot showing the median and interquartile range (IQR) for Acute Panic Inventory (API) scores in the Clinical and Comparison Groups (N = 30) at Time 1, Time 2, and Time 3. The boxes represent the IQR, with the median indicated by a horizontal line. Whiskers extend to values within 1.5 times the IQR, and asterisks denote significant differences (p <.05).

API ratings were significantly different across the three times, χ^2^(2) = 28.404, p <.001. *Post-hoc* analysis with Wilcoxon signed-rank tests was conducted with a Bonferroni correction applied, resulting in a significance level set at Alpha = p < 0.017. The median API ratings were 1.0 at Time 1, 7.5 at Time 2, and 2.5 at Time 3. A significant increase was seen between Time 1 and Time 2, (Z = -4.401, p <.001). A significant decrease was observed between Time 2 and Time 3, (Z = 3.235, p =.001). There were no significant differences between the participants’ baseline API measures and those taken at time 3 following the CO_2_ challenge, (Z = -.1651, p>.017). **Figure 3** displays a box plot of participants’ API scores measured at baseline (Time 1), following the CO_2_ challenge (Time 2), and after the CFI and CO_2_ challenge (Time 3).

**Figure 4:**
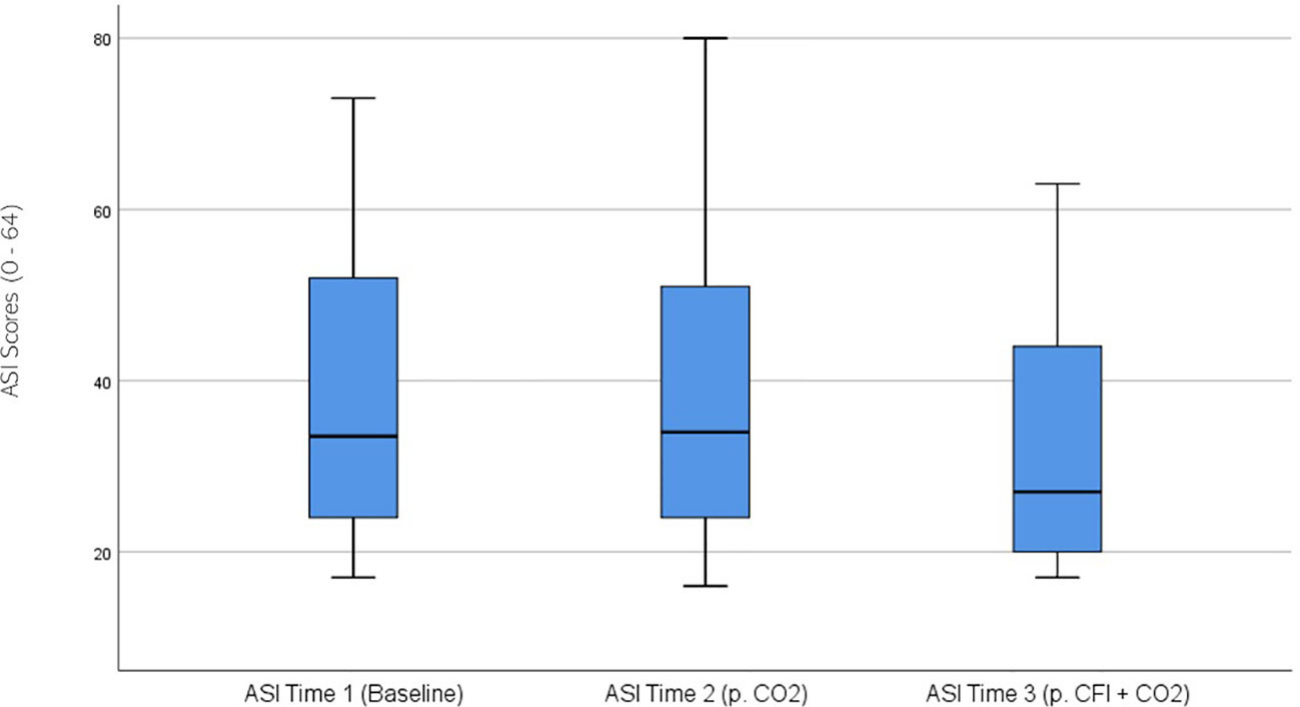
Box plot showing the median and interquartile range (IQR) for Anxiety Sensitivity Index (ASI) scores in the Clinical and Comparison Groups (N = 30) at Time 1, Time 2, and Time 3. The boxes represent the IQR, with the median indicated by a horizontal line. Whiskers extend to values within 1.5 times the IQR, and asterisks denote significant differences (p <.05).

ASI ratings were significantly different across the three times, χ^2^(2) = 17.741, p < 0.01. *Post-hoc* analysis with Wilcoxon signed-rank test was conducted with a Bonferroni correction applied, resulting in a significance level set at Alpha = p <.016. The median ASI ratings were 33.5 at Time 1, 34 at Time 2, and 27 at Time 3. No significant difference was observed between baseline and Time 2, (Z = 0.520, p >.017), whilst a significant decrease was noted between Time 2 and Time 3, (Z = 2.654, p =.008). A significant decrease was also observed between baseline and Time 3, (Z = -3.019 p =.003). **Figure 4** displays a box plot of participants’ ASI scores measured at baseline (Time 1), following the CO_2_ Challenge (Time 2), and after the CFI and CO_2_ Challenge (Time 3).

**Figure 5:**
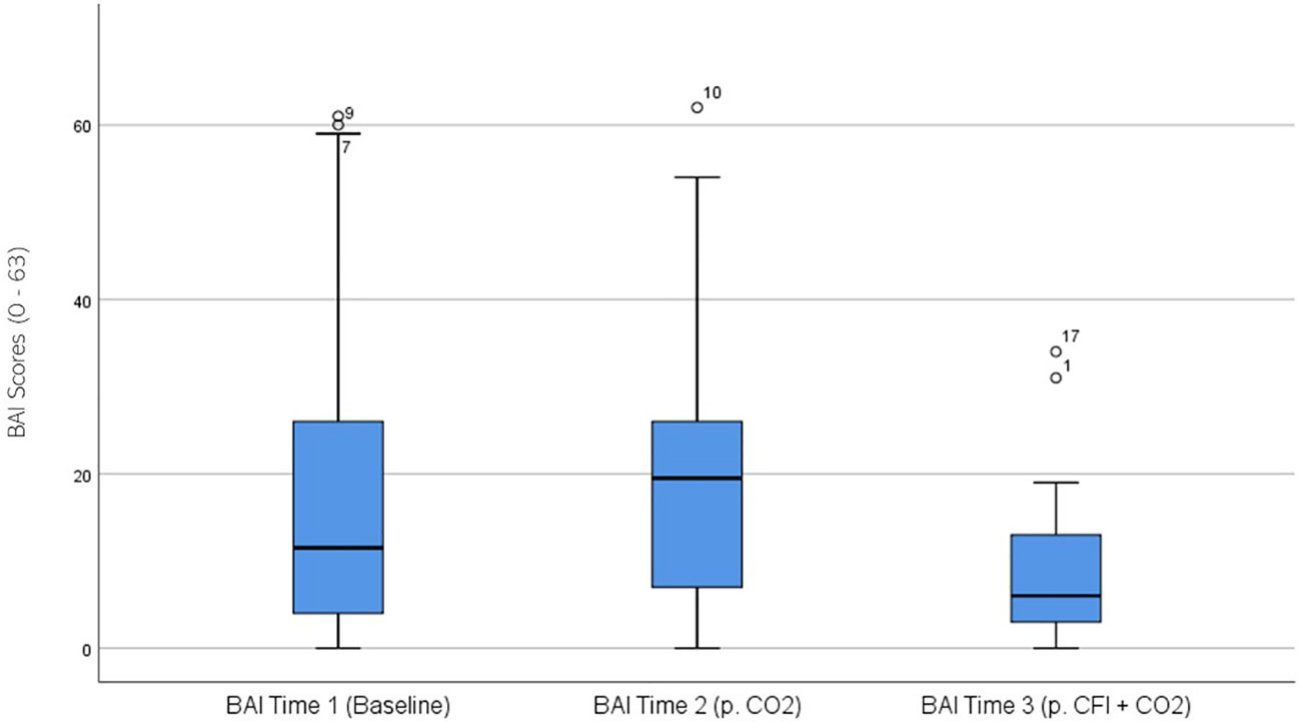
Box plot showing the median and interquartile range (IQR) for Beck Anxiety Inventory (BAI) scores in the Clinical and Comparison Groups (N = 30) at Time 1, Time 2, and Time 3. The boxes represent the IQR, with the median indicated by a horizontal line. Whiskers extend to values within 1.5 times the IQR, and asterisks denote significant differences (p <.05).

BAI ratings were significantly different across the three times, χ^2^(2) = 14.131, p = .001. *Post-hoc* analysis with Wilcoxon signed-rank test was conducted with a Bonferroni correction applied, resulting in a significance level set at Alpha =p <.017. The median BAI ratings were 11.51 at Time 1, 19.5 at Time 2, and 6.0 at Time 3. No significant difference was noted between Time 1 and Time 2 (Z = -1.211, p >.017). A significant decrease was seen between Time 1 and Time 3, (Z = -3.607, p <.001). A significant decrease was observed between Time 1 and Time 3, (Z = -2.849, p = .004). **Figure 5** displays a box plot of participants’ BAI scores measured at baseline (Time 1), following the CO_2_ Challenge (Time 2), and after the CFI and CO_2_ challenge (Time 3).

**Figure 6:**
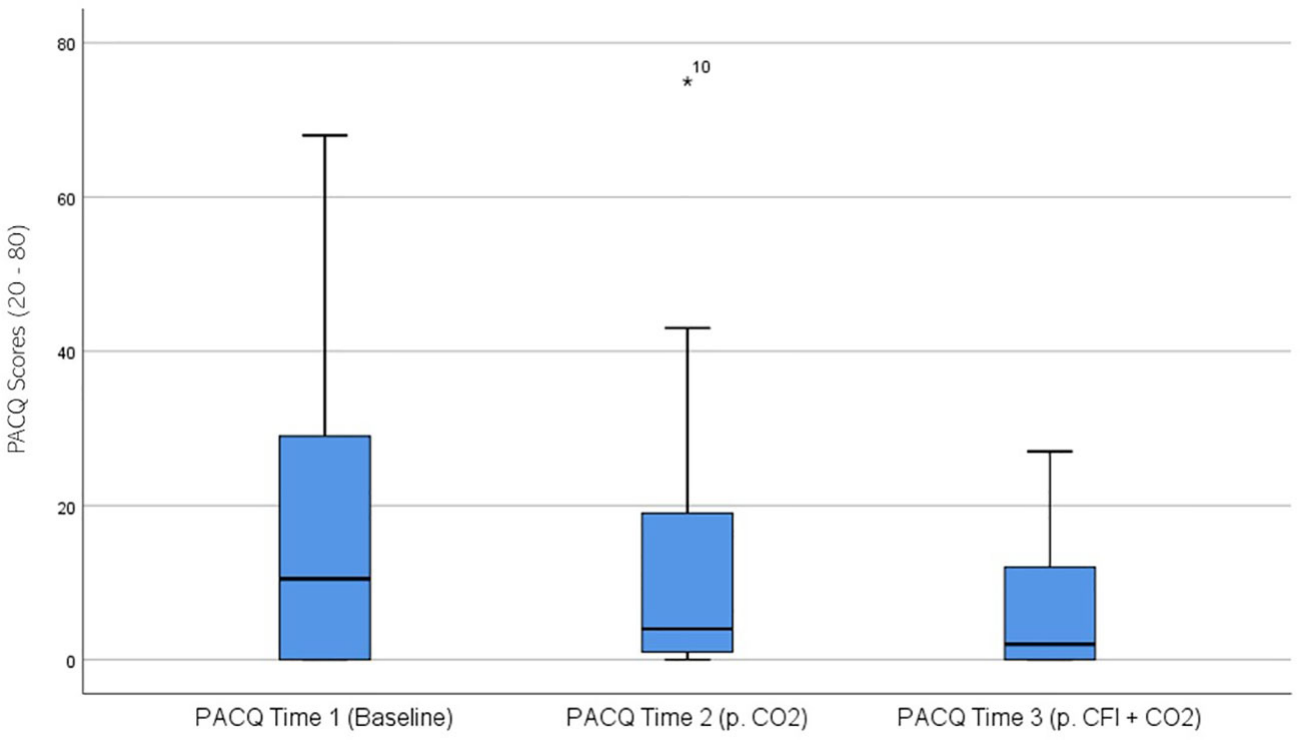
Box plot showing the median and interquartile range (IQR) for Panic Cognitions Questionnaire (PACQ) scores in the Clinical and Comparison Groups (N = 30) at Time 1, Time 2, and Time 3. The boxes represent the IQR, with the median indicated by a horizontal line. Whiskers extend to values within 1.5 times the IQR, and asterisks denote significant differences (p <.05).

PACQ ratings were significantly different across the three times, χ^2^(2) = 12.896, p = .002. *Post-hoc* analysis with Wilcoxon signed-rank test was conducted with a Bonferroni correction applied, resulting in a significance level set at Alpha = p <.017. The median PACQ ratings were 10.5 at Time 1, 4.0 at Time 2, and 2.0 at Time 3. No significant differences were observed between baseline and time 2, (Z = 2.234, p >.017), and Time 2 and Time 3 (Z = -2.218, p >.017). A significant decrease was observed between Time 1 and Time 3, (Z = 3.495, p <.001). **Figure 6** displays a box plot of participants’ PACQ scores measured at baseline (Time 1), following the CO_2_ challenge (Time 2), and after the CFI and CO_2_ challenge (Time 3).

**Figure 7:**
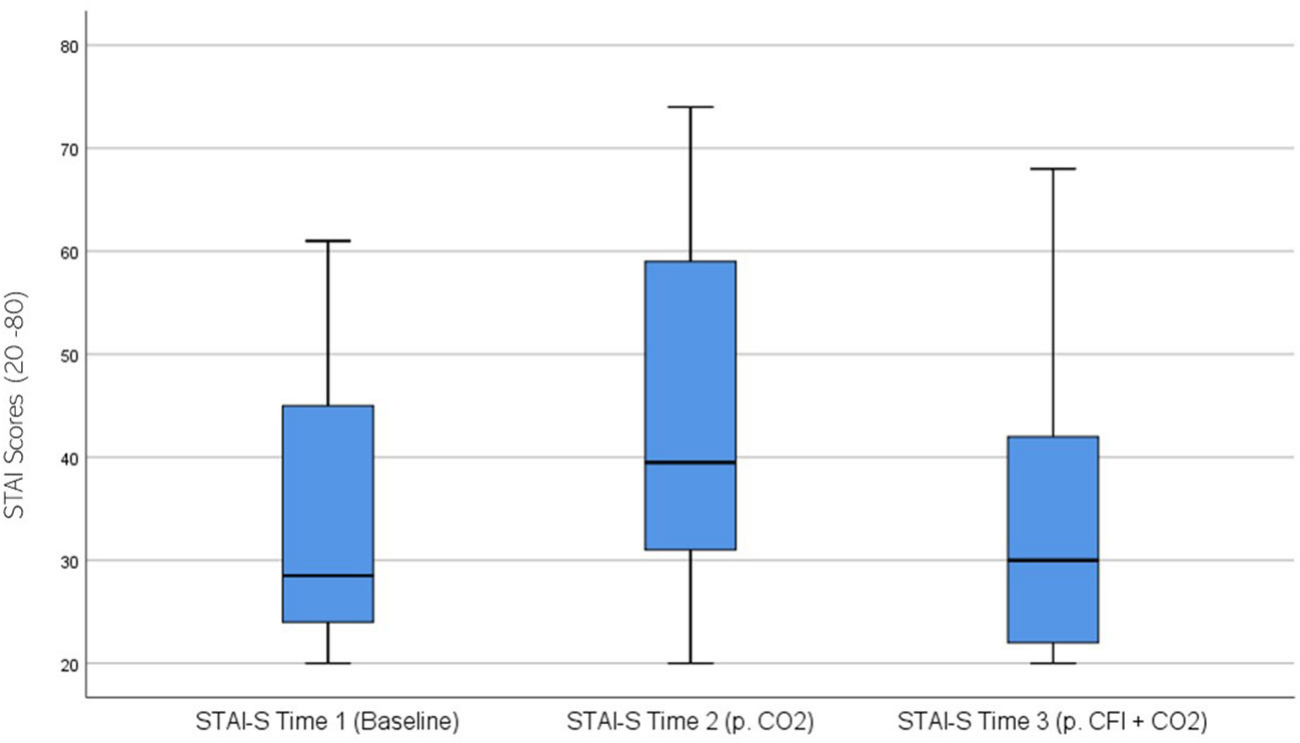
Box plot showing the distribution of State-Trait Anxiety Inventory (STAI) scores for the Clinical and Comparison Groups (N = 30) at Time 1, Time 2, and Time 3. The boxes represent the interquartile range (IQR), with the median indicated by a horizontal line. Whiskers extend to values within 1.5 times the IQR, and asterisks denote significant differences (p <.05).

STAI ratings were significantly different across the three time periods, χ^2^(2) = 23.078, p < 0.01. *Post hoc* analysis with Wilcoxon signed-rank tests was conducted with a Bonferroni correction applied, resulting in a significance level set at Alpha = p <.017. The median STAI ratings were 28.5 at Time 1, 39.5 at Time 2, and 30.0 at Time 3. There was a significant increase between the participants’ baseline STAI measures and those taken at time 2, following the CO_2_ challenge, (Z = -.4.159, p <.001). A statistically significant decrease was seen between Time 2 and Time 3, (Z = -3.305, p = .001). There was no significant difference between Time 1 and Time 3, (Z = -0.833, p >.017). **Figure 7** displays a box plot of participants’ STAI-S scores measured at baseline (Time 1), following the CO_2_ challenge (Time 2), and after the CFI and CO_2_ challenge (Time 3).

**Figure 8:**
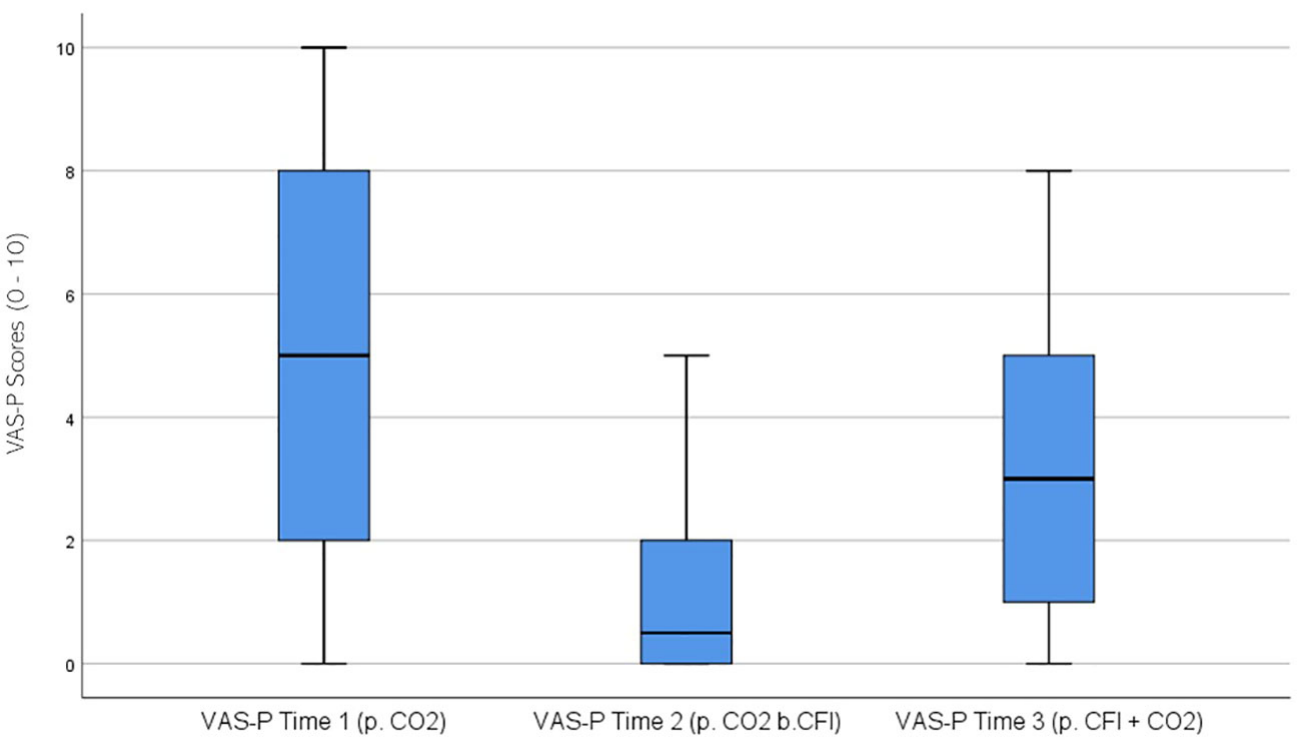
Box plot showing the median and interquartile range (IQR) for Visual Analog Scale – Participant (VAS-P) scores in the Clinical and Comparison Groups (N = 30) at Time 1, Time 2, and Time 3. The boxes represent the IQR, with the median indicated by a horizontal line. Whiskers extend to values within 1.5 times the IQR, and asterisks denote significant differences (p <.05).

VAS Participant (VAS-P) ratings were significantly different across the three times, χ^2^(2) = 28.055, p < 0.01. *Post-hoc* analysis with Wilcoxon signed-rank test was conducted with a Bonferroni correction applied, resulting in a significance level set at Alpha = p <.017. The median VAS-P ratings were 5.0 at Time 1, 0.5 at Time 2, and 3.0 at Time 3. Significant decreases were observed between baseline and Time 2, (Z = -4.363, p <. 001), and between Time 2 and Time 3, (Z = -3.782 p <. 001). Furthermore, a significant decrease was seen between Time 1 and Time 3, (Z = -2.801, p = .005). **Figure 8** displays a box plot of participants’ VAS-P scores measured at baseline (Time 1), following the CO_2_ challenge (Time 2), and after the CFI and CO_2_ challenge (Time 3).

**Figure 9:**
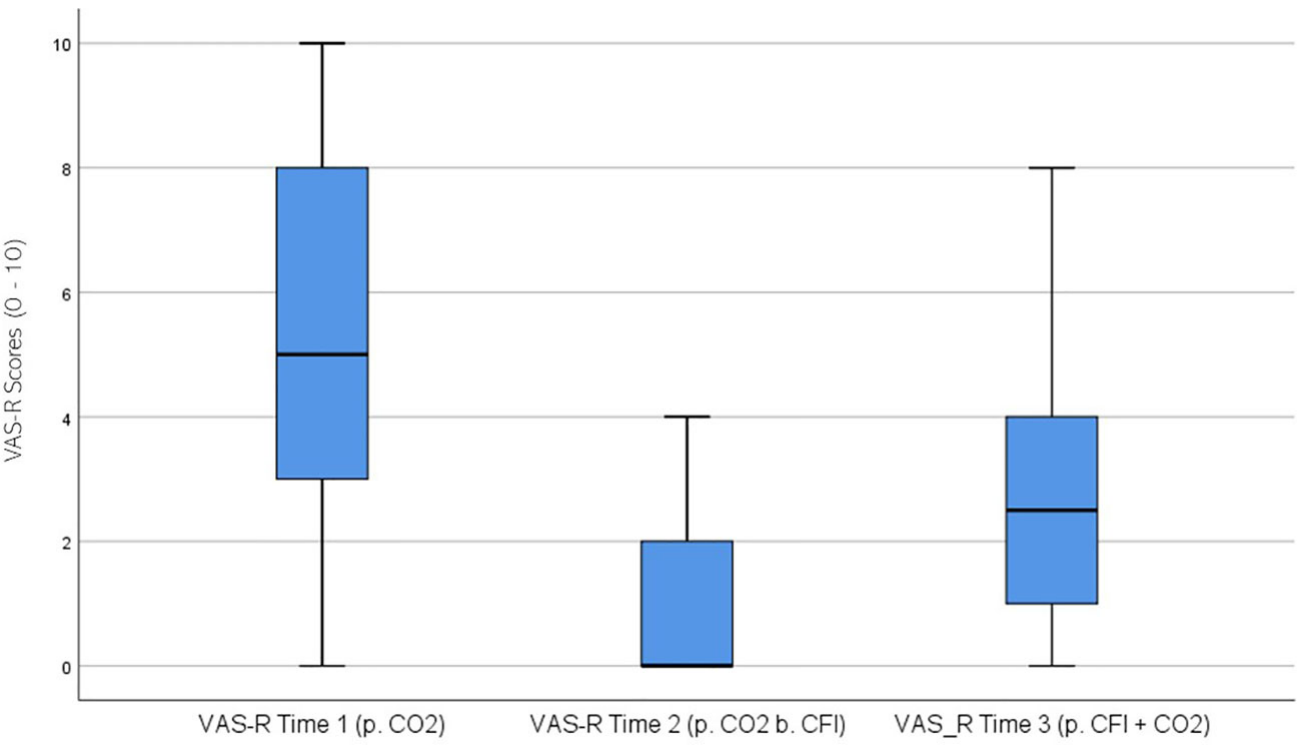
Box plot showing the median and interquartile range (IQR) for Visual Analog Scale – Researcher (VAS-R) scores in the Clinical and Comparison Groups (N = 30) at Time 1, Time 2, and Time 3. The boxes represent the IQR, with the median indicated by a horizontal line. Whiskers extend to values within 1.5 times the IQR, and asterisks denote significant differences (p <.05).

VAS Researcher (VAS-R) ratings were significantly different across the three times, χ^2^(2) = 39.086, p < 0.01. *Post-hoc* analysis with Wilcoxon signed-rank test was conducted with a Bonferroni correction applied, resulting in a significance level set at Alpha =p <.017. The median VAS-R ratings were 5.0 at Time 1, 0 at Time 2, and 2.5 at Time 3. A significant decrease was seen between baseline and Time 2, (Z = -4.462, p <.001), and between Time 2 and Time 3, (Z = 4.178 p <.001). Furthermore, there was a significant decrease between Time 1 and Time 3, (Z = -3.506, p <.001). **Figure 9** displays a box plot of VAS-R scores measured at baseline (Time 1), following the CO_2_ challenge (Time 2), and after the CFI and CO_2_ challenge (Time 3).

## Discussion

The principal findings of this study were that there were no differences in heart rate and respiration rate within or between groups in response to the CO_2_ challenge. Following the CO_2_ challenge, the Clinical Group was observed to have more elevated anxiety and panic symptoms, as reported by the anxiety measures in comparison to the Comparison Group. Furthermore, a significant reduction in anxiety symptomatology was observed in both the Clinical and Comparison Groups following the cold facial immersion (CFI). The results of this study are consistent with the finding of the preliminary study (12).

It was expected that the CFI task would reduce CO_2_ sensitivity, as evidenced by heart rate and respiration rate reductions following the subsequent CO_2_ challenge in Clinical and Comparison participants. The findings did not provide support for this hypothesis as there was no significant difference found in participants’ heart rate and respiration rate between Time 2 (CO_2_ challenge) and Time 3 (CFI and CO_2_). However, the results examining the effects of the CFI task (prior to the administration of CO_2_) indicate a significant reduction in heart rate for both Clinical and Comparison participants. The clinical group had a larger reduction in heart rate between Time 2 and Time 3 compared to the comparison group suggesting that the CFI may be a useful intervention for the treatment of PD. In line with the findings of Kyriakoulis et al. (12), these results indicate that CFI has anxiolytic effects. However, despite its powerful response, it cannot be inferred that one single administration of CFI can alter one’s sensitivity to CO_2_.

The study found that there was a significant reduction noted between Time 1 (baseline pre-measures) and Time 3 (CFI followed by CO_2_) on the following anxiety measures Anxiety Sensitivity Index (ASI), State and Trait Anxiety Inventory (STAI), Panic Attack Cognitions Questionnaire (PACQ), Beck Anxiety Inventory (BAI), Visual Analog Scale completed by the participant (VAS-P) and researcher (VAS-R), with the exception of Acute Panic Inventory (API) for the both the Clinical and Comparison Groups. Hence, the CFI task led to broad improvements in anxiety-related symptoms, especially in cognitive, emotional, and perceived anxiety. Furthermore, significant decreases in anxiety symptoms were reported from Time 2 (CO_2_) to Time 3 (CFI + CO_2_) on the following measures; API, ASI, BAI, STAI, VAS-R, and VAS-P, with the exception of the PACQ which was not found to change significantly between Time 2 and 3. A possible explanation for this finding is that Comparison Group participants did not experience panic cognitions in response to the CO_2_ challenge. As non-parametric analyses were used to assess the effects of the CFI on CO_2_, this did not allow for a comparison between groups across the different time points. Whilst the Comparison Group had minimal symptoms overall in response to the CO_2_ challenge, they did report a reduction in symptoms and calming effects in response to the CFI task. The Clinical Group reported a decrease in anxiety symptoms, following exposure to the CFI task on all anxiety measures including the ASI, API, STAI, PACQ, BAI, VAS-R and VAS-P. Hence frequent exposure to the CFI tasks and to breath-holding may be able to reduce CO_2_ sensitivity in individuals with PD over time.

Practicing breath-holding and activating the DR through freediving and CFI regularly has been known to have trained effects, subsequently reducing CO_2_ sensitivity (14, 15). Reports in the literature have suggested that a blunted hypercapnic response and prolonged breath-hold times have been associated with the benefits of trained effects and repeated apneas (25–29). Goosens et al. (30) in their study found that experienced divers displayed a decreased behavioral response to CO_2_ as compared to clinical participants with PD and healthy comparisons. This finding is also consistent with the research of Earing et al. (31), who found that experienced divers possess a lower ventilatory response to CO_2_ suggesting a strong adaptation of central CO_2_ sensitivity. Through breath-holding training, the body of a freediver adapts to prolonged periods of low oxygen (hypoxia) and high CO_2_ (hypercapnia). This leads to reduced sensitivity to CO_2_, allowing better control during deep dives where oxygen and CO_2_ levels fluctuate. Over time, the body becomes accustomed to higher CO_2_ levels without triggering the typical urge to breathe.

Whilst the current study was unable to identify whether one single administration of the CFI task can alter CO_2_ sensitivity, it appears promising as an intervention given that it was able to significantly reduce self-reported anxiety symptoms and reduced heart rate for both Clinical and Comparison participants.

### Study limitations

Limitations of this study included recruitment challenges, and the extensive list of exclusion criteria for individuals to be eligible to participate in the study, which limited the sample size and matching of participants. As a result of recruitment challenges, researchers invited five participants from a preliminary study (12) who had expressed interest in participating in future research in this area. In order to ameliorate recruitment challenges participants with intermittent use of benzodiazepines, anxiolytics and painkillers were allowed to participate in the current study and we extended the age from 55 years of age to 60 years of age, in an attempt to recruit more participants.

Despite efforts to match participants and recruit non-university students as comparisons, achieving this proved challenging. Therefore, the results may not accurately reflect a typical population. Some participants reported challenges, such as disliking the taste of CO_2_ gas, feeling anxious during the task, and struggling to inhale deeply. Although every effort was made, a few participants were unable to hold their breath for the required four seconds. Given that this is required for the test to be considered a valid, participants must inhale at least 80% of their vital capacity of CO_2_ to experience the full effects of the CO_2_ challenge (8).

A further limitation is that both the CO_2_ challenge and the CFI task elicit a brief response. A drawback to this may be that participants may not accurately report what they experience as by the time they complete the self–report anxiety measures minutes later, their symptoms may have dissipated. In the current study, cold facial immersion bradycardia reached a peak within 20 to 30 seconds, which is consistent with previous research (25, 32). Reports in the literature suggest that lower water temperatures (0˚C - 10˚C) lead to an increase in minute ventilation and a more pronounced bradycardic response compared to warmer water (32, 33). The effectiveness and extent of the activation of the DR can vary based on factors such as water temperature and the duration of immersion (34). However, acute panic responses were less responsive to change, indicating a potential need for more targeted treatment in that area.

Other methodological considerations for future studies investigating the CFI and the DR include improving the provocation of the CO_2_ challenge, the continuous data acquisition during the experimental design, and matching participants for age and gender, as well as including the VAS-P and VAS-R measures at baseline before any condition as a means of comparison.

### Considerations for future research

Research has established a clear link between respiratory disorders and PD (35–41). Future studies should investigate the differences between respiratory and the non-respiratory clinical groups to assess how each responds to the provocation methods (i.e., breath-hold tasks, 35% CO_2_ challenge and CFI task). Specifically, future research is needed to investigate the time course for the development of the trained effects, determining how often and how many apnea exposures are required (26). This area of inquiry will help clarify benefits of breath-hold training and CFI as treatments for PD. Understanding the physiological adaptations of apnea training and freediving techniques, such as the activation of the DR, may also contribute to the development of innovative treatments for panic and anxiety symptoms (14).

### Implications and future directions

This study opens several avenues for future research on the role of the DR and breath-hold training in managing PD. Given that individuals with PD often exhibit heightened sensitivity to CO_2_ and autonomic dysfunction, exploring the effects of controlled breath-hold tasks, like CFI, on both physiological and psychological symptoms could inform novel therapeutic interventions. Specifically, future research should investigate the long-term effects of CFI practice and other apnea-based techniques on anxiety sensitivity and panic symptoms (42, 43). Understanding how the DR contributes to anxiety regulation may also help integrate breath-hold exercises into cognitive-behavioral therapy (CBT) for PD, leveraging DR adaptations to address anxiety sensitivity and maladaptive beliefs linked to panic-related issues (42, 43).

While freediving techniques have shown promise in regulating autonomic function, the optimal frequency and duration of breath-hold tasks needed to produce lasting benefits remain unclear. Future studies should systematically examine different apnea exposure protocols to determine their effects on vagal tone, heart rate variability, and emotion regulation (33, 44). Additionally, identifying individual differences in response to apnea training could help determine which subgroups of PD patients may benefit most from such interventions.

Beyond the therapeutic potential of breath-hold training, the CFI task itself could serve as a valuable diagnostic and intervention tool for assessing physiological and psychological responses to CO_2_ challenges. By activating the DR, CFI may reduce heart rate and alleviate physiological and cognitive symptoms of panic, potentially decreasing CO_2_ sensitivity. Clinicians could use CFI to observe changes in heart rate, respiratory rate, and self-reported anxiety symptoms, gaining insights into a patient’s sensitivity to physiological arousal and their susceptibility to panic attacks. Moreover, heart rate variability (HRV) could be a useful biomarker for studying potential changes in panic and anxiety symptoms following CFI and breath hold training. Future research should explore how CFI could be integrated into psychoeducation or as part of a behavioral experiment, to help individuals challenge and correct maladaptive beliefs about bodily sensations associated with panic. Previous research indicates that addressing anxiety sensitivity can help reduce panic attacks and implementing behavioral experiments that activate the DR could help patients confront feared sensations and reframe their interpretations of physiological symptoms (45). Barlow et al. (46–48) highlighted the efficacy of cognitive restructuring and behavioral experiments in significantly reducing anxiety symptoms, further supporting the potential of DR-based techniques in treatment.

While this study has focused on the implications of DR activation and CFI for PD, future research should investigate the potential benefits of DR activation and CFI for other anxiety-related disorders, such as generalized anxiety disorder or social anxiety disorder, where heightened sensitivity to bodily sensations is a common feature. This could expand the application of these techniques beyond PD and into other areas of anxiety treatment.

## Conclusion

This study found that the CFI task effectively reduced anxiety and panic symptoms triggered by the CO_2_ challenge. Although, there were no significant differences between the Clinical and Comparison Groups, the findings suggest that the CFI task may still be beneficial for individuals with PD. One of the most distressing symptoms for those with PD is tachycardia (increased heart rate), which can amplify feelings of anxiety and panic. By activating the DR through cold exposure, it is possible to reduce the heart rate, which in turn may help alleviate these symptoms. The reduction in heart rate is thought to contribute to an anxiolytic effect on the autonomic nervous system, helping to counter the physiological arousal that often accompanies anxiety and panic. Therefore, the activation of the DR, which is easily achieved through methods like CFI and cold moisture (e.g., ice packs), could serve as an accessible and effective treatment for panic disorder and other anxiety disorders s. Whilst these findings are promising, further research is needed to explore the long-term effects and optimal use of DR activation in clinical settings. Specifically, investigating the precise mechanisms, the frequency of its application, and its broader therapeutic potential would be critical for advancing its role in anxiety treatment. anxiolytic effects of DR activation.

## Data Availability

The raw data supporting the conclusions of this article will be made available by the authors, without undue reservation.
